# Asthenosphere dynamics based on the H_2_O dependence of element diffusivity in olivine

**DOI:** 10.1093/nsr/nwaa278

**Published:** 2020-11-12

**Authors:** Tomoo Katsura, Hongzhan Fei

**Affiliations:** Bayerisches Geoinstitut, University of Bayreuth, Germany; Center for High Pressure Science and Technology Advanced Research, China; Bayerisches Geoinstitut, University of Bayreuth, Germany

## Abstract

The oceanic asthenosphere shows two enigmatic features: low viscosity and high electrical conductivity. Their origins gather wide attention, but remain unsolved. Recent self-diffusivity measurements as a function of H_2_O content in olivine demonstrated that the H2O-incorporation in olivine cannot soften the asthenosphere, but it enhances the ionic conductivity, and causes the high-conductivity anomaly.

The oceanic asthenosphere is one of the most enigmatic regions in the Earth's interior. The presence of seismic low-velocity zones implies high softness at the top of the asthenosphere, which may cause smooth plate motion. Although the high temperature and relatively low pressure of the asthenosphere may cause this softness, it is unclear whether smooth plate motion originates from the temperature and pressure dependence alone. The significant high softness was previously interpreted as creep enhancement by partial melting. However, this interpretation was later dismissed, because the melts are gravitationally unstable in the asthenosphere and will migrate rapidly due to the high melt permeability in mantle rocks [1]. Asthenosphere softening has recently been interpreted by the incorporation of H_2_O in mantle minerals. Olivine is the dominant mineral in the asthenosphere, and is well known to incorporate up to several thousand wt. ppm H_2_O in the crystal structure [2]. High-resolution deformation experiments suggest that H_2_O incorporation largely enhances olivine creep rates [3]. Because the H_2_O incorporated in olivine is immobile, the interpretation based on H_2_O incorporation may be more reasonable than the partial melting hypothesis.

Another problem with the topmost asthenosphere is the high electrical conductivity layer (HCL) near the East Pacific Rise [4], which disappears away from the ridge as the geotherm is lowered. A special effect is therefore believed to enhance conductivity near the ridge. Because H_2_O incorporation can cause proton conduction by the migration of free protons (${\rm{H}}_{\rm{i}}^\cdot$), free-proton conductivity is hypothesized as a means to interpret the HCL [4].

The above hypotheses based on H_2_O incorporation in olivine are, however, deficient for the following reasons. For creep, the H_2_O-enhancement rate was determined only nominally in previous deformation experiments [5]. This is because the samples in those deformation experiments were saturated with free-H_2_O, and the H_2_O-fugacity (${f_{{{\rm{H}}_2}{\rm{O}}}}$) was controlled by the confining pressure. In this way, the effect of H_2_O on creep rates can be arbitrarily obtained by assuming a pressure dependence, which is poorly constrained. Although the creep rates were previously concluded to be proportional to $f_{{{\rm{H}}_2}{\rm{O}}}^{1.2}$ [3], this value was deduced by assuming an activation volume of 38 cm^3^/mol [5], which is unrealistic, because it is close to the molar volume of olivine. Moreover, strain rates in the deformation apparatus are approximately 10 orders of magnitude higher than those in the asthenosphere, which may cause different creep mechanisms. For conductivity, firstly, the magnitude of proton conduction is too small to interpret the HCL [4]. Secondly, the geophysical observation indicates a large temperature dependence of the HCL, whereas proton conduction is relatively insensitive to temperature [4].

To overcome the problems with the deformation experiments, we adopted a different strategy. Climb-controlled dislocation creep, which dominates in the asthenosphere, is controlled by diffusion of the slowest species: Si (slowest) and O (second slowest). We therefore measured the Si and O self-diffusivity of olivine as a function of ${C_{{{\rm{H}}_2}{\rm{O}}}}$, pressure, and temperature, and estimated the ${C_{{{\rm{H}}_2}{\rm{O}}}}$ dependences of the creep rate based on diffusivity [6–10]. Pipe diffusion has been suggested to also play an essential role in dislocation creep [11]. We therefore measured both Si lattice and grain-boundary diffusivity, because the H_2_O effect on pipe diffusivity should be between these diffusivity types. The diffusion experiments were conducted under quasi-hydrostatic conditions, which are much closer to the asthenospheric conditions than those in the deformation experiments. Because the ${C_{{{\rm{H}}_2}{\rm{O}}}}$ and pressure ranges can be largely and independently controlled in diffusion experiments, the ${C_{{{\rm{H}}_2}{\rm{O}}}}$ and pressure dependences can be determined independently.

We also measured Mg self-diffusivity as a function of ${C_{{{\rm{H}}_2}{\rm{O}}}}$, pressure and temperature [12]. The conduction mechanism appearing at the highest temperatures in olivine is ionic conduction driven by the migration of ${\rm{V}}_{{\rm{Mg}}}^{2 - }$, which should be controlled by Mg self-diffusion. The high-temperature occurrence of the HCL suggests an essential role of ${\rm{V}}_{{\rm{Mg}}}^{2 - }$ ionic conductivity rather than the ${\rm{H}}_{\rm{i}}^ \cdot $ proton conduction. It is very difficult to measure ionic conductivity as a function of ${C_{{{\rm{H}}_2}{\rm{O}}}}$ because H_2_O easily escapes from olivine under the required high-temperature conditions. On the other hand, the high-temperature ionic conductivity can be evaluated by Mg self-diffusivity measured at relatively low temperatures.

The experimental results are shown in Table 1 and Fig. 1a–c. The ${C_{{{\rm{H}}_2}{\rm{O}}}}$ exponents of Si and O lattice self-diffusivity are 0.32 ± 0.07 and 0.05 ± 0.06, respectively. These values are much smaller than suggested by the deformation experiments (1.2). Because the ${C_{{{\rm{H}}_2}{\rm{O}}}}$ exponent of Si grain boundary self-diffusivity is identical to that of Si lattice diffusivity within error (0.26 ± 0.07), that of pipe diffusivity should be identical to these values. If the creep rate is proportional to these ${C_{{{\rm{H}}_2}{\rm{O}}}}$ dependences, the effects of H_2_O on asthenospheric dynamics will be negligibly small [7], contrary to the conclusion from the deformation experiments [3]. The deformation experiments demonstrated a clear difference in creep rate between H_2_O-free and H_2_O-saturated olivine [3]. These observations indicate that trace amounts of H_2_O (<10 wt. ppm) may drastically decrease the creep strength. Larger amounts of H_2_O, however, will not increase the creep rate according to our results, and the H_2_O variation in the upper mantle will not change the asthenospheric dynamics.

**Figure 1.**
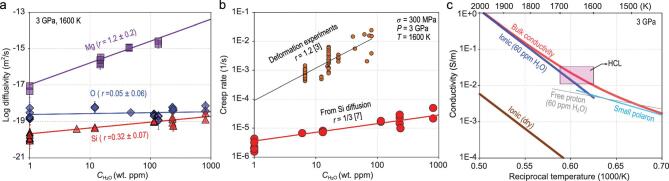
(a) Lattice self-diffusivity of Si (red) [7], O (blue) [8] and Mg (violet) [11] of olivine as a function of H_2_O content after pressure and temperature corrections to 3 GPa and 1600 K, respectively, corresponding to topmost asthenosphere conditions. (b) Comparison of creep rates in olivine obtained by deformation experiments (orange) [3] and estimated from Si self-diffusivity (red) [7]. (c) Electrical conductivity of olivine with various mechanisms. The pink rectangle shows conditions of the HCL [4]. Cyan, small polaron [4]; brown, ionic conductivity under dry conditions [4]; blue, ionic conductivity with 60 wt. ppm H_2_O [9]; gray, free proton conduction with 60 wt. ppm H_2_O [4]; red, sum of small polaron and ionic conductivity with 60 wt. ppm H_2_O [13], which is used to interpret the HCL [4].

**Table 1.** Parameters of Si, O and Mg self-diffusivity and ionic conductivity of olivine.

	Si lattice	Si grain boundary	O lattice	Mg lattice	Ionic conductivity
${C_{{{\rm{H}}_2}{\rm{O}}}}$ exponent	0.32 ± 0.07 [7]	0.26 ± 0.07 [10]	0.05 ± 0.06 [8]	1.2 ± 0.2 [12]	1.3 ± 0.2 [13]
Activation energy (kJ/mol)	410 ± 30 [6]	220 ± 30 [10]	400 ± 30 [8]	250 ± 30 [12]	250 — 405 [13]
Activation volume (cm^3^/mol)	1.7 ± 0.4 [6]	4.0 ± 0.4 [10]	−3.9 ± 1.2 [9]	4.3 ± 0.3 [12]	4.3 ± 1.0 [13]
H_2_O range (wt. ppm except for Si grain boundary diffusion)	<1–800	<1–53.3 μm wt. ppm	<1–800	<1–350	20–580
Temperature range (K)	1600, 1800	1200–1600	1600, 1800	1100–1300	1450–2180
Pressure range (GPa)	1–13	1–13	1–13	1–13	2–10

In contrast to Si and O, Mg self-diffusivity has a large ${C_{{{\rm{H}}_2}{\rm{O}}}}$ dependence, i.e. the ${C_{{{\rm{H}}_2}{\rm{O}}}}$ exponent is 1.2 ± 0.2 [12]. This suggests a significant H_2_O enhancement of the ionic conductivity. Very recently, we succeeded in a direct measurement of ionic conductivity in olivine as a function of ${C_{{{\rm{H}}_2}{\rm{O}}}}$, pressure, and temperature, proving a strong H_2_O enhancement of the ionic conductivity [13]. The identical ${C_{{{\rm{H}}_2}{\rm{O}}}}$, pressure, and temperature dependences between Mg self-diffusivity and ionic conductivity (Table 1) suggest the essential role of Mg diffusion in ionic conductivity. As shown in Fig. 1c, the sum of the small polaron and ionic conductivity with 60 wt. ppm of H_2_O can reproduce the conductivity of the HCL. In contrast, the magnitude of proton conduction is too small to account for the HCL with realistic H_2_O contents in the depleted MORB mantle [4].

The above arguments indicate that H_2_O enrichment is not required to interpret the asthenosphere dynamics. The effects of H_2_O on mineral properties are sometimes overestimated and overlooked by direct measurements. The overestimation may have occurred because the ${C_{{{\rm{H}}_2}{\rm{O}}}}$ dependence was determined with various ${C_{{{\rm{H}}_2}{\rm{O}}}}$ samples by varying the pressure without correct knowledge of the pressure dependence. This was the case with creep experiments under water-saturated conditions. The oversight occurred with the high-temperature phenomena, because the H_2_O effects are difficult to observe at high temperatures due to H_2_O loss from the samples, as was the case with high-temperature electrical conductivity. A vital mineral property obtained by one method must be examined in an independent way to obtain a correct understanding of mantle dynamics. The measurement of self-diffusivity is a useful method to examine H_2_O effects on physical properties originating in atomic diffusion such as rheology and electrical conductivity.
